# Prospective Randomized Controlled Trial of the Impact of Olfactory Training on Cognitive and Emotional Function in Individuals with Parkinson Disease

**DOI:** 10.1016/j.arrct.2025.100497

**Published:** 2025-07-24

**Authors:** Yunxiao Dou, Siyu Qian, Yichen Zhao, Yan Tan, Yanxin Zhao

**Affiliations:** a Department of Neurology, Shanghai Tenth People’s Hospital, Tongji University School of Medicine, Shanghai.; b Department of Neurology, Jing'an District Zhabei Central Hospital, Shanghai.; c Department of Neurology, Shanghai Eighth People’s Hospital, Shanghai, China.; d Department of Neurology, Qingpu Branch of Zhongshan Hospital, Shanghai, China.

**Keywords:** Anxiety, Cognition, Depression, Memory, Nonmotor symptoms, Olfactory training, Parkinson disease, Rehabilitation

## Abstract

**Objective:**

To investigate the clinical effects of olfactory training (OT) on olfactory function and related nonmotor symptoms in patients with Parkinson disease (PD).

**Design:**

Randomized controlled trial with a duration of 6 months of follow-up.

**Setting:**

A tertiary hospital providing neurology rehabilitation services in China.

**Participants:**

Of 81 initially recruited PD patients, 70 completed the study (N=70, 35 per group after randomization: OT group n=35, control n=35). All participants met inclusion criteria and received standard medical care. No dropouts were because of adverse effects.

**Interventions:**

The intervention group received OT using a standardized protocol. The training involved daily exposure to specific odorants for 6 months. The control group did not receive OT.

**Main Outcome Measures:**

Primary outcomes included changes in olfactory test scores, memory and cognition scores (assessed using neuropsychological tests), and depressive and anxiety scale scores. These were measured before and after the 6-month intervention.

**Results:**

After 6 months, the intervention group exhibited significant improvements in olfactory test scores (threshold-discrimination-identification score: t=3.839, *P*<.01, Cohen’s d=0.649), memory (memory quotient: t=2.597, *P*<.05, Cohen’s d=0.439) and cognition scores (Mini-Mental State Examination: z=−2.791, *P*<.01, rank-biserial correlation (*r*)=−0.330 and Montreal Cognitive Assessment: t=2.626, *P*<.05, Cohen’s d=0.444), depressive (Hamilton Depression Scale: z=−3.601, *P*<.001, *r*=−0.425 and Patient Health Questionnaire-9: z=−2.396, *P*<.05, *r*=−0.29) and anxiety (Hamilton Anxiety Scale: z=−3.049, *P*<.01, *r*=−0.36 and Generalized Anxiety Disorder-7: z=−2.849, *P*<.01, *r*=0.336) scale scores compared to the control group, showing moderate effects, respectively. The increase in memory scale scores was positively correlated with the increase in olfactory scores (Spearman *r*=0.415, *P*<.05). Statistical analysis was performed using SPSS 27.0 software, with *P* values indicating significance.

**Conclusions:**

The OT has beneficial effects on olfactory function and related nonmotor symptoms in patients with PD. The 6-month training led to significant improvements in memory, cognition, depression, and anxiety. Further studies are needed to determine the long-term effects and optimal duration of OT for patients with PD.

Parkinson disease (PD) is a progressive, chronic neurodegenerative disorder characterized by both motor and early, often overlooked nonmotor symptoms.^1^ Nonmotor symptoms such as olfactory dysfunction and cognitive-affective symptoms can be seen in the initial phases of PD.^2^ Although current drug treatments can control motor symptoms, these nonmotor symptoms have a key role in causing severe disability and reducing the quality of life.^3^, ^4^, ^5^

Increasing clinical evidence supports the notion that olfactory training (OT) can enhance the olfactory function of patients with olfactory dysfunction, including those with PD.^6^, ^7^, ^8^, ^9^, ^10^ Consequently, OT may represent a vital treatment option for individuals requiring rehabilitation of their sense of smell. Nonetheless, most research on the outcomes of OT has primarily concentrated on olfactory function. Given the existence of direct neural pathways linking the olfactory system to the amygdala-hippocampal complex, the amygdala’s connectivity with olfactory and learning and memory circuits positions it as a plausible mediator of OT effects.^11^ Consequently, we hypothesize that the beneficial effects of olfactory therapy may extend to memory and cognition beyond olfaction in patients with PD.

Projections from the olfactory cortex extend to the hippocampus and orbitofrontal cortex to form a odor-limbic-cognitive loop.^12^^,^^13^ Within this circuit, the hippocampus is involved in affective behavioral changes.^14^^,^^15^ The orbitofrontal cortex acts as a hub for assigning emotional valence to odors and coordinating odor-guided decision-making.^16^^,^^17^ This intricate network underlies the influence of olfactory sensation on emotions. Furthermore, numerous studies have explored specific patterns of olfactory stimulation.^18^^,^^19^ These findings collectively suggest that specialized olfactory experiences can shape emotions-related neural circuitry. Building upon this evidence, our secondary hypotheses propose that structured OT may extend these effects to ameliorate depressive and anxiety symptoms.

The subjects with PD were recruited into this single-center, prospective, controlled, nonblinded study, which aimed to examine whether potentially improved olfactory function could affect memory, cognition, and emotions in patients with PD via 6-month OT.

## Methods

### Subjects

The sample size was determined by using G*Power 3.1 software.^20^^,a^ In order to obtain the power of 0.80 with an alpha level set to 0.05 to detect a moderate effect size of dz=0.5 within the difference between 2 dependent means (matched pairs), the projected sample size was at least 34 subjects.

Participants were consecutively recruited in the Parkinson disease clinic of the Department of Neurology in Shanghai Tenth People’s Hospital between August 2023 and October 2024. They were divided into 2 groups randomly with the help of a computer-generated random array (allocation ratio 1:1), where the subjects in the intervention group were treated with OT under the original PD-related treatment, and the subjects in the control group were not exposed to OT. The grouping results corresponding to the computer-generated random number list were hidden in sealed, opaque numbered envelopes, which were opened consecutively after obtaining informed consent. The researchers who performed the random assignment sequence and determined the eligibility of the participants were not the same person.

Inclusion criteria included: (1) subjects aged ≥18 years old; 2) patients who have been diagnosed with PD based on the clinical diagnostic criteria set by the Movement Disorder Society,^21^ and those who had been taking consistent antiparkinsonian medication for a minimum of 4 weeks before beginning the study and continued to do so throughout the study.

The exclusion criteria included: (1) patients meeting the diagnostic criteria for dementia according to the Diagnostic and Statistical Manual of Mental Disorders-V; 2) individuals meeting the diagnostic criteria for Alzheimer’s disease, vascular dementia, frontotemporal dementia, and other forms of dementia; (3) patients meeting the diagnostic criteria for Parkinson plus syndrome, secondary parkinsonism, and other parkinsonian syndromes; 4) patients with systemic diseases involving the heart, liver, lung, kidney, endocrine system, and blood that can lead to cognitive impairment.

**Fig 1:**
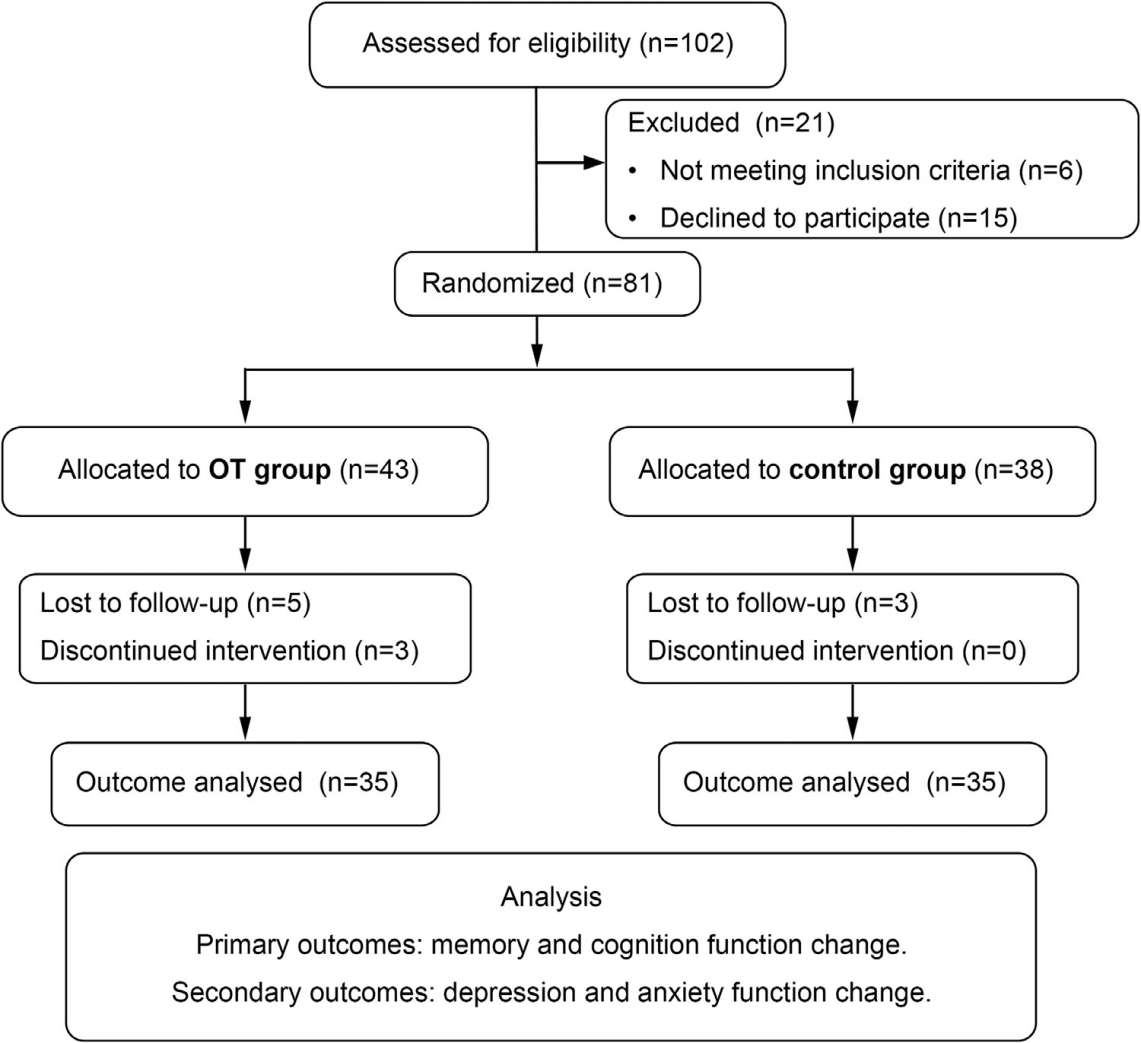
Flow diagram of the study**.**

All the patients got the detailed information of the experiment, and they all provided written consent. The study protocol was approved by the Ethics Committee of Shanghai Tenth People’s Hospital. (Approved number: SHSY-IEC-5.0/22K111/P01), and it conformed to the provisions of the Helsinki Declaration revised in 2013. The study was also registered in the Chinese Clinical Trials Registry (Registration number: ChiCTR2200062414). We used the flow diagram and checklist in figure 1 with reference to CONSORT 2010 statement.^22^

### Baseline assessment

The clinical data of patients, including age, sex, course of the disease, education, Hoehn and Yahr stage, and antiparkinsonian medication, were collected.

### Procedure

Four sniff bottles were used in the set-up for the olfactory test, each containing a different scent for the distribution of 4 odors. The scents included phenylethyl alcohol (rose scent), eucalyptol (eucalyptus scent), citronella (lemon scent), and eugenol (clove scent).^23^ As standard OT,^23^ the patients were required to sniff each of the 4 scents separately 2 times a day for at least 40 seconds.

### Outcome measures

The primary outcomes of the study were memory and cognition function change in patients with PD. The secondary outcomes included improvement in depression and anxiety symptoms. In addition, all patients underwent an olfactory test and PD rating scale before and after OT treatment. Relevant methods were shown in the supplemental appendix S1 (available online only at http://www.archives-pmr.org/).

### Statistical analysis

SPSS 27.0^b^ software was used to perform all statistical analyses. Continuous variables were described using the mean ± standard deviation (SD), whereas categorical variables were presented as percentages. The normality of the distribution of continuous variables was assessed using the Kolmogorov–Smirnov test. Two independent samples *t* test or paired *t* test was used for between-group comparisons. The Mann–Whitney *U* test or paired Wilcoxon test was used for the variables with nonnormal distribution. The chi-square test was used for counting data. The association between 2 variables was investigated by the *r*correlation of Pearson or Spearman. *P*<.05 indicated statistical significance. Primary analyses followed the intention-to-treat principle, including all randomized participants in their originally assigned groups regardless of protocol adherence or dropout.

## Results

Table 1 shows the demographic and clinical data of all subjects. Among 81 PD patients initially enrolled in the study, 11 dropped out (8 patients were lost to follow-up, 3 patients did not complete the OT treatment plan), resulting in 70 patients who completed all the scale tests. Baseline characteristics were balanced between OT (n=35) and control (n=35) groups: age: OT 65.8±7.3 vs control 67.8±8.4 years (*t*=0.302, *P*=.585). Sex: male proportion was comparable (OT 54.3% vs control 45.7%, χ²=0.514, *P*=.473). PD duration: OT 9.3±4.6 vs control 9.5±4.5 months (Z=−0.431, *P*=.666). Hoehn and Yahr stage: stage 1-2: OT 54.3% vs control 57.1%, stage 2.5-3: OT 45.7% vs control 42.9% (χ²=0.094, *P*=.759); Education level: No significant difference (χ²=2.235, *P*=.569); Medication regimens: Equally distributed (χ²=1.107, *P*=.775), with levodopa monotherapy being most common (OT 45.7% vs control 34.3%). Data presented as mean ± SD or n (%).

**Table 1:** Descriptive statistics of patients of groups.

Descriptive statistics of patients of two groups	OT Group(n=35)	Control Group(n=35)	F/Z/x2	*P* Value
Age, y	65.83±7.282	67.77±8.405	0.302	.585
Sex, male (%)	19 (54.3)	16 (45.7)	0.514	.473
Duration, mo	9.31±4.575	9.51±4.520	−0.431	.666
Education			2.235	.569
Primary (%)	2 (5.7)	3 (8.6)		
Junior (%)	18 (51.4)	12 (34.3)		
Senior (%)	10 (28.6)	14 (40.0)		
University and above (%)	5 (14.3)	6 (17.1)		
Hoehn and Yahr stage			0.094	.759
1-2 (%)	19 (54.3)	20 (57.1)		
2.5-3 (%)	16 (45.7)	15 (42.9)		
Antiparkinsonian medication			1.107	.775
Levodopa (%)	16 (45.7)	12 (34.3)		
Levodopa + Dopamine agonist (%	7 (20.0)	9 (25.7)		
Levodopa + Anticholinergic (%)	6 (17.1)	6 (17.1)		
Levodopa + MAOB inhibitor (%)	6 (17.1)	8 (22.9)		

Data given as mean ± SD.

### Olfactory function

**Fig 2:**
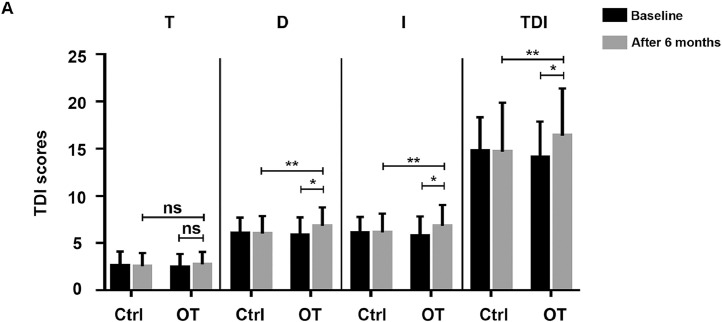
Evaluation of olfactory function with the TDI score. Olfactory function was evaluated by the TDI score (comprehensive score of threshold, discrimination, and identification abilities) at baseline and after the 6-month training period in the training and control group. Data are presented as the mean ± SD, ns>.05, *P<.05, **P<.01.

A Sniffin' Sticks olfactory test was administered to all patients both before and after treatment, and the results were assessed based on the composite threshold-discrimination-identification score. As shown in figure 2, there was no significant difference in the TDI score between the training group and the control group during the evaluation before the training. After OT, the TDI score of the training group was significantly higher than that of the control group (*t*=2.160, *P*<.05). Also, the training group showed improvement as they progressed through the training program (TDI: *t*=3.839, *P*<.01, Cohen’s d=0.649), whereas no improvement was found in controls (*t*=2.004, *P*=.053); 11% of the training group participants showed an olfactory function that was improved **≥**5.5 points, indicating significant clinically meaningful improvement,^24^ whereas no patients in the control group had such improvement. In 3 subparts of olfactory ability, discrimination (z=−3.040, *P*<.01, *r*=−0.359) and identification (*t*=3.218, *P*< .01, Cohen’s d=0.544) scores increased in trained PD patients, but no improvement was observed in threshold ability (*t*=1.381, *P*=.176, Cohen’s d=0.233). In short, the training group showed a specific improvement in their measured olfactory function after undergoing OT. In particular, significant improvements were found in odor discrimination and identification ability.

### Memory

**Fig 3:**
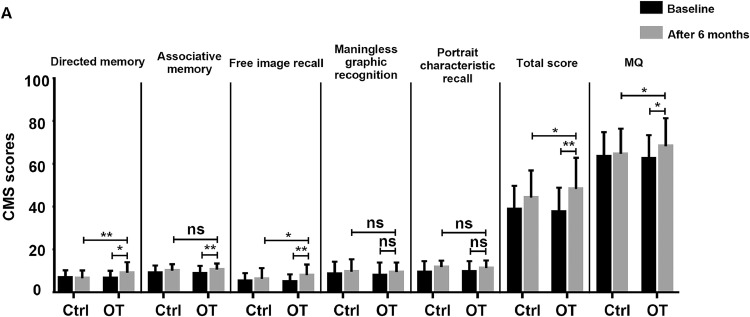
Memory function was assessed by clinical memory scale (CMS). The 5 test scores, total scale score, and MQ of CMS were employed to assess the memory function of subjects at baseline and after 6 months in the training and control group. Data are presented as the mean ± SD, ns>.05, *P<.05, **P<.01.

The cognitive memory system included a directed memory test and an associates test, both used to assess verbal memory. Additionally, nonverbal memory was evaluated through tests such as free image recall, meaningless graphic recognition, and portrait characteristic recall.^25^ MQ was converted from the total scale score according to age and educational background. In figure 3, our results showed no significant difference in the total MQ scores and MQ between the training group and controls before the training (*P*>.05). After OT, the total MQ scores (*t*=3.418, *P*<.01) and MQ (*t*=2.010, *P*<.05) of the *t* training group were significantly higher than that of the control group. In addition, trained PD patients experienced a significant increase in their total memory quotient scores (*t*=2.400, *P*<.05) and MQ (*t*=2.597, *P*<.05) compared to baseline. There were no significant results in the control group before and after (*P*>.05). Therefore, OT significantly improves memory function in PD patients, including verbal memory and nonverbal memory.

### Cognitive function

**Fig 4:**
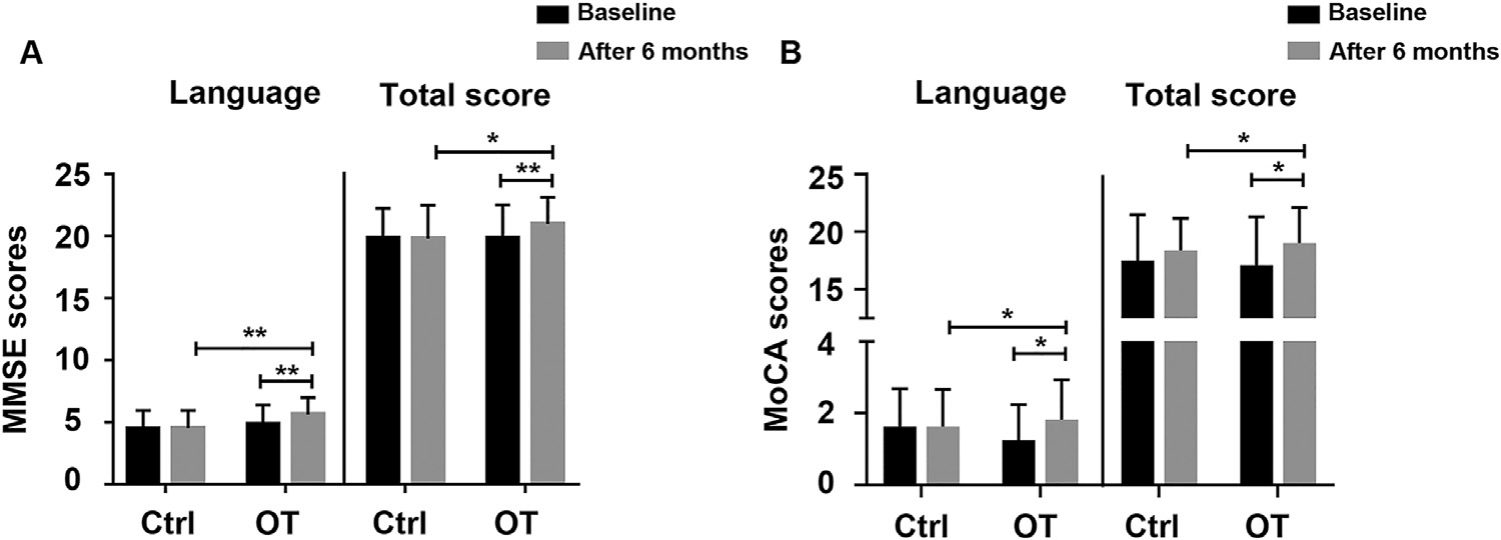
The MMSE and MoCA were used to estimate general cognitive function. (A) The MMSE total score and language score showed the cognitive function change of subjects in the training and control group before and after 6 months. (B) The MoCA total score and language score of the training group and control group are shown. Data are presented as the mean ± SD, *P<.05, **P<.01.

In this work, both MMSE scores and MoCA were used to estimate general cognitive function. In figure 4, our results showed that the total score of MMSE (z=−2.110, *P*<.05) and MoCA (*t*=2.167, *P*<.05) of the training group were significantly higher than that of the control group after OT. Also, the total score of MMSE (z=−2.791, *P*<.01) and MoCA (*t*=2.626, *P*<.05) of the training group improved during the course of training, whereas this was not observed in controls. In MMSE **(**fig 4A), trained PD patients experienced a significant increase in their language score compared to baseline (z=−3.166, *P*<.01) and compared to the control group (z=−2.653, *P*<.01), with no significant results in other domains. In MoCA **(**fig 4B**)**, patients with PD who underwent training showed a substantial improvement in their language skills (z=−2.138, *P*<.05) compared to baseline and compared to the control group (z=−2.266, *P*<.05). There were no significant results in other domains. Thus, OT had a positive effect on cognition, especially in the language domain.

### Mood

**Fig 5:**
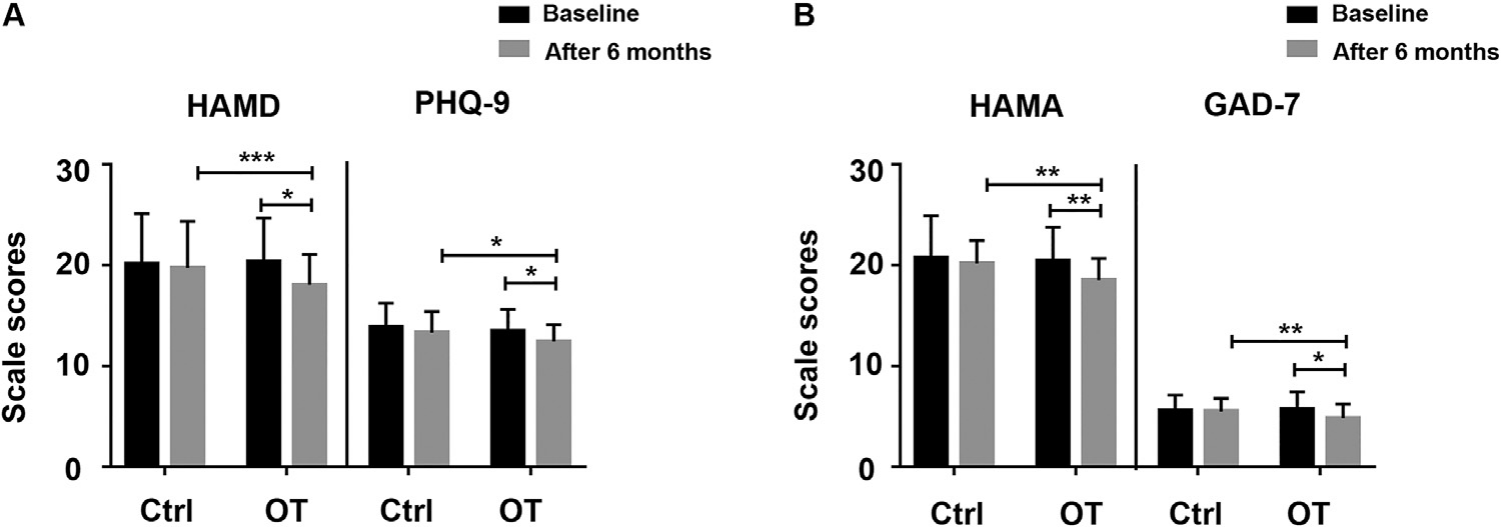
Changes in depression and anxiety symptoms were tested by emotion scales. (A) Depression symptom of subjects in the training and control group before and after 6 months was tested by the HAMD, PHQ-9. (B) Anxiety symptom was assessed using the HAMA, 7-item GAD-7. Data are presented as the mean ± SD, *P<.05, **P<.01, ***P<.001.

In depression scales in figure 5A, we found that the total score of HAMD (z=−3.601, *P*<.001) and PHQ-9 (z=−2.396, *P*<.05) in the training group improved during the course of training; the total score of HAMD (z=−2.448, *P*<.05) and PHQ-9 (z=−2.515, *P*<.05) in the training group was significantly higher compared to the control group after OT. Considering anxiety scales in figure 5B, trained PD patients experienced a significant increase in the total score of HAMA (z=−3.049, *P*<.01) and GAD-7 (z=−2.849, *P*<.01) compared to baseline. Compared with the control group, both HAMA (z=−2.600, *P*<.01) and GAD-7 (z=−2.007, *P*<.05) scores of the training group were significantly higher than those of the control group after OT. In summary, OT significantly improved depression and anxiety in PD patients.

### PD rating analysis

**Fig 6:**
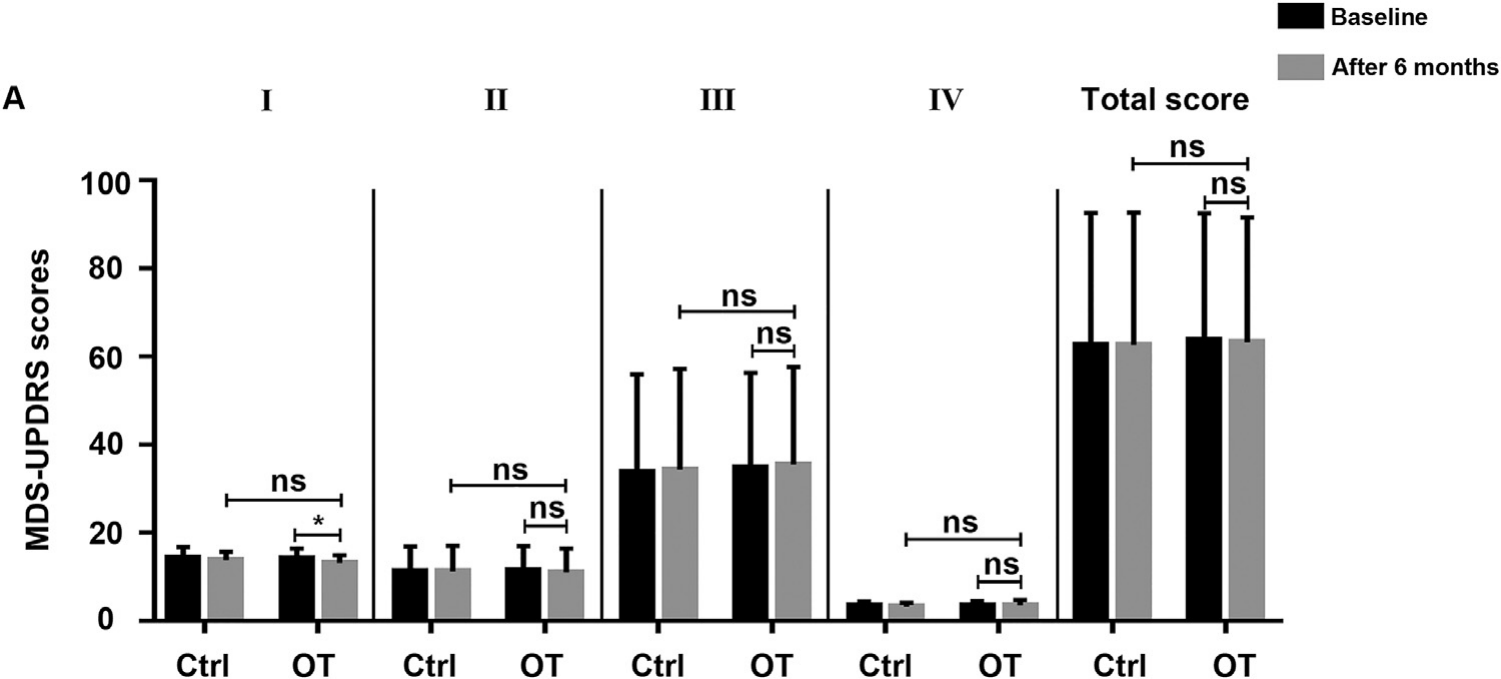
PD-related symptoms were assessed by MDS-UPDRS. The total MDS-UPDRS score and 4 parts scores were calculated for each participant before and after 6 months. Data are presented as the mean ± SD, ns>.05, *P<.05.

Part III focused on motor exploration. The evaluation of the total score and the MDS-UPDRS-Ⅲ score after the training showed no significant difference between the training group and controls in figure 6. However, the MDS-UPDRS- I (nonmotor experiences of daily living) score of the training group was higher than that of the control group after OT (z=−2.063, *P*<.05). In brief, OT had a positive effect on nonmotor symptoms other than motor function.

### Correlation

We initially hypothesized that training olfactory perception would also affect other related nonmotor processing areas. We examined the associations between the increase in the training subjects’ TDI score and improvement in their memory, cognition, and emotion scale scores by *r* correlation of Pearson or Spearman. As shown in table 2, intercorrelations between the changes (Δ) in olfactory and other domains revealed that the ΔTDI score was positively related with ΔMQ (Spearman’s rho=0.415, *P*<.05), which suggested that the improvement of olfactory function was positively correlated with the improvement of memory. Therefore, there was a relationship between training-induced improvements in olfactory sense and memory in PD patients.

**Table 2:** Spearman’s correlations for the relationships between changes (Δ) in olfactory function and memory, cognitive, and emotions scores.

Variable	Correlations	ΔMQ	ΔMMSE	ΔMoCA	ΔHAMD	ΔPHQ-9	ΔHAMA	ΔGAD-7	
ΔTDI	Spearman’s rho	0.415*	0.258	0.160	0.037	0.170	0.319	−0.008	
	*P* value	0.013	0.135	0.359	0.832	0.329	0.062	0.965	

TDI, threshold-discrimination-identification score.

⁎ Correlation is significant at the 0.05 level (2-tailed).

### Safety

No serious adverse events were observed.

## Discussion

The present study was conducted to explore the clinical effects of OT and whether potentially improved olfactory function could affect other related nonmotor symptoms in patients with PD after OT intervention. Our results indicated that the beneficial effects of OT extended beyond olfaction, having a positive effect on memory, cognitive function, and depressive and anxiety symptoms. After 6 months, the intervention group exhibited significant improvements in olfactory test scores (TDI: *t*=3.839, *P*<.01, Cohen’s d=0.649), memory (MQ: *t*=2.597, *P*<.05, Cohen’s d=0.439) and cognition scores (MMSE: z=−2.791, *P*<.01, rank-biserial correlation (*r*)=−0.330 and MoCA: *t*=2.626, *P*<.05, Cohen’s d=0.444), depressive (HAMD: z=−3.601, *P*<.001, *r*=−0.425 and PHQ-9: z=−2.396, *P*<.05, *r*=−0.29) and anxiety (HAMA: z=−3.049, *P*<.01, *r*=−0.36 and GAD-7: z=−2.849, *P*<.01, *r*=0.336) scale scores compared to the control group, showing moderate effects, respectively. The increase in memory scale scores was positively correlated with the increase in olfactory scores (Spearman’s rho=0.415, *P*<.05).

OT has been proven to enhance the sense of smell in humans, and is now recognized as a beneficial treatment for individuals experiencing smell loss after infection or trauma.^23^^,^^26^^,^^27^ Our results indicated that the beneficial effects of OT extended beyond olfaction, having a positive effect on cognitive function in patients with PD. Recent studies have underscored the significant role of the amygdala in the pathogenesis of PD, particularly concerning cognitive impairment and olfactory dysfunction.^28^, ^29^, ^30^ These findings provide a compelling rationale for considering the amygdala’s contribution to the benefits observed after OT in our study. In the research conducted by Ay et al,^28^ the early involvement of the amygdala in the caudo-rostral progression of Lewy pathology in PD was highlighted, revealing a significant difference in total amygdala volume among patients with PD with varying degrees of cognitive impairment compared to healthy controls. Additionally, the research contributes to this body of knowledge by establishing an association between dopamine availability, amygdala volume, and cognitive dysfunction in PD.^30^ This study underscores the intricate relationship between the amygdala, dopaminergic systems, and cognitive decline in PD. Our findings, in the context of these studies, suggest that OT may exert its therapeutic effects, in part, by modulating amygdala function. However, we acknowledge that our study did not directly assess amygdala function or volume, and our discussion is speculative. Future studies incorporating neuroimaging to directly examine the amygdala’s role during OT could provide valuable insights into the neural mechanisms underlying the therapeutic effects of OT on cognitive and emotional functions in patients with PD.

In this work, we concluded that OT significantly improved memory of patients with PD; the improvement of olfactory function was positively correlated with memory improvement. Although researchers have not studied the function of OT on memory in patients with PD, they have discussed that the cortical substrates of olfaction broadly overlap with those involved in memory. A study highlighted a connection between the sense of smell and verbal memory,^31^ the other demonstrated a strong connection between the sense of smell and memory, both of which rely on the hippocampus and medial orbitofrontal cortex.^32^ In the latter study, researchers examined the idea that this connection is because of common neural bases. This association could be attributed to the similar development of the olfactory and hippocampal systems. Therefore, we speculated that there would be notable alterations in the connectivity of the brain network among patients with PD after training. Enhancing the functional connections in these areas could potentially lead to the improvement of memory function in individuals with PD.

Moreover, our results indicated that OT significantly improved depression and anxiety in patients with PD. Because of the partial overlap in the brain structures associated with emotions and those related to smell, particularly involving the limbic system and prefrontal regions,^13^ this complex network forms the basis for the effects of olfactory sensation on emotion, in turn providing an anatomy and physiology basis for the effect of OT on emotions in patients with PD. It is interesting to note that in a longitudinal study, OT significantly improved general well-being and depression in older people with impairments.^33^ Moreover, the data indicate that the positive effect of OT on emotional well-being may be attributed to its influence on the connection between crucial olfactory structures and areas such as the amygdala and orbitofrontal cortex,^34^ which play a significant role in the manifestation of symptoms related to depression and anxiety.^33^ In this study, there is a correlation between olfactory improvement and memory improvement, but not with other cognitive or emotional domains. We suspect that memory may respond faster to OT because of hippocampal neurogenesis, while frontal-executive functions require longer plasticity.^35^

### Study limitations

There are limitations to the current study. First, it is a single-center study with a small sample size. More multicenter studies with a much larger sample size are warranted. Second, OT can be demanding for both patients and health care providers over an extended period of time. Adhering to the established protocol of OT requires a significant level of discipline. Future studies should determine whether the observed improvement in nonmotor symptoms is temporary or continues beyond the training period. In addition, ‘PD-related biomarkers’ and ‘MRI’ were exploratory aims in the registry but excluded in the final analysis because of technical feasibility

## Conclusions

Overall, OT has beneficial effects on olfactory function and related nonmotor symptoms in patients with PD. The 6-month training led to significant improvements in memory, cognition, depression, and anxiety. Further studies are needed to determine the long-term effects and optimal duration of OT for patients with PD.

## Suppliers

a. G*Power, version 3.1; Heinrich Heine University Düsseldorf.
b. SPSS, version 27.0; IBM.

## Disclosure

The investigators have no financial or nonfinancial disclosures to make in relation to this project.

## Appendix Supplementary materials

Image, application 1

## Footnotes

Supplementary material associated with this article can be found, in the online version, at doi:10.1016/j.arrct.2025.100497.
