# Supplementary material for: Prospective Randomized Controlled Trial of the Impact of Olfactory Training on Cognitive and Emotional Function in Individuals with Parkinson Disease
Source: Arch Rehabil Res Clin Transl. 2025 Jul 24;7(4):100497. doi: 10.1016/j.arrct.2025.100497 (PMC12750363; doi:10.1016/j.arrct.2025.100497)
Supplement: Supplementary file 1 [file mmc1.docx]

Supplemental Appendix S1:

Materials and Methods

**2.1 Subjects**

Given the overt nature of OT, participants were necessarily unblinded. However, outcome assessors were blinded to group allocation during all tests. Randomization codes were concealed from statisticians until database lock. This partial blinding approach aligns with CONSORT guidelines for non-pharmacological trials [1]. All adverse events (AEs) were monitored throughout the trial. If an AE occurred, the study coordinator documented the event and assessed severity using CTCAE v5.0 criteria. Participants received appropriate clinical care (e.g., symptom management). Unless the AE was intervention-related and severe (Grade ≥3), participants continued in the trial. All AEs were reported to the ethics committee within 24 hours for serious AEs (SAEs). Among 81 PD patients initially enrolled, 11 dropped out (8 lost to follow-up, 3 non-adherence), leaving 70 for final analysis (35 OT, 35 control). No OT-related AEs occurred.

The trial protocol was modified during implementation to prioritize outcomes with higher clinical relevance to olfactory training (OT). While motor function (MDS-UPDRS-III) was initially a primary outcome, interim analysis showed no significant changes, whereas olfactory (TDI) and memory (CMS) improvements were robust. Mood scales (HAMD/HAMA) were added as secondary outcomes to align with recent evidence. Biomarker and MRI analyses were omitted due to logistical constraints. All adjustments were approved by the ethics committee and did not affect the trial’s validity. We regret that our trial had to be terminated in October 2024, but this decision was made after careful consideration of trial feasibility and the rational use of resources.

**2.3 Procedure**

Our approach aligns with Haehner et al [2], who combined diaries and caregiver checks in PD patients. To ensure rigorous adherence monitoring in the OT group, we implemented a triangulated verification system. Participants maintained daily logs of odor exposure (timing/duration) through patient diaries. For patients with cognitive concerns (MMSE ≤26), caregivers co-signed logs and provided independent reports. Besides, Biweekly phone calls cross-checked compliance and addressed protocol questions. Considering longer OT had larger effects in neurodegenerative disorders [2, 3], we decided to perform 6 months olfactory training. To reduce the influence of prefrontal fatigue on advanced function, we used the order of smell-cognition-emotion [4]. Breaks were mandated every 30 minutes, and alternate test forms minimized learning effects.

**2.4 Outcome measures**

Olfactory testing

‘Sniffin’ Sticks' smell test (Burghart, Wedel, Germany) was used as previously described. The exam is divided into three sections: odor thresholds (T), odor discrimination (D), and odor identification (I). The sum of the scores from these subsections determine the TDI score, which has a maximum of 48 points. In brief, the odor thresholds test is accomplished by a so-called "gradient test procedure". The initial perceived concentration of the odor was determined by the dilution gradient used to find the pen that was just enough to distinguish between the odorless pen (blank) and the odorant. The odor discrimination test was performed based on the comparison of the odors of three olfactory pens in groups. In one group, two pens had the same odor (non-target) and the other had a different odor (target). The subjects were tasked with pointing out which pens in a group of pens smelled different from the others, and 16 groups of pens were compared throughout the experiment. The odor identification test is used to evaluate the ability to recognize everyday odors and is accomplished by choosing the smell of a sniffing pen from a four-option cue card. The curative effect was evaluated according to the total TDI score before and after treatment. An increase in TDI of 5.5 points or threshold score of 2.5 points, or an identification score of 3 points was considered effective [5, 6].

The study participants were all tested in a quiet and well-ventilated setting. The sticks were positioned 2 cm away from the nostrils and presented at intervals of 20-30 seconds. Consistency was maintained as the same researcher conducted all the tests.

**Memory function**

The memory function was evaluated using a clinical memory scale (CMS) adopted by the Institute of Psychology of the Chinese Academy of Sciences. The CMS consists of five tests: direct memory, associative memory, recognition of meaningless graphics, free image recall, and recall of portrait characteristics. Upon completion of the tests, the raw scores were adjusted based on age and education level as per the guidelines provided. These adjusted scores were then combined to calculate the total scale score, which was subsequently converted into a memory quotient (MQ) following the guidelines. The memory function was categorized into different levels based on the MQ results: a score of 130 or above indicated super-excellence, 120–129 denoted excellence, 110–119 signified upper level performance, 90–109 indicated moderate level performance, 80–89 represented lower level performance, 70–79 suggested poor performance, and an MQ below 70 indicated very poor memory function [7].

**Cognitive measures**

The Mini-Mental State Examination (MMSE) and Montreal Cognitive Assessment (MoCA) were employed to assess overall cognitive function. In the Chinese population, the Chinese adaptation of the MMSE was utilized for dementia detection [8]. The MMSE evaluates five areas: orientation in terms of place and time, registration, attention and calculation, recall, language, and visual construction [9].

In the current study, the MoCA version translated into Beijing dialect from English was administered, which consists of seven cognitive domains: visuospatial/executive function, naming, attention, abstraction, language, delayed memory, and orientation [10]. A score below 26 on the MMSE and MoCA has been utilized as a cutoff point to indicate evidence of cognitive impairment [11, 12].

In the current study, we administered a battery of cognitive tests that included MoCA and MMSE to evaluate cognitive function. The Memory Scale was chosen to provide a detailed assessment of various memory components, while the MoCA and MMSE were selected for their established sensitivity in detecting cognitive impairments in clinical settings. Although MoCA and MMSE both assess global cognitive status, MoCA is designed to be more sensitive to mild cognitive impairments, particularly in the domains of executive function and attention, which are common in early stages of neurodegenerative diseases. MMSE, on the other hand, is a widely used and well-validated tool for general cognitive screening. By using both MoCA and MMSE, we aimed to capture a comprehensive view of cognitive function while increasing the robustness of our assessment.

**Emotion tests**

Emotional symptoms were measured using the 21-item Hamilton Depression Scale (HAMD), 9-item Patient Health Questionnaire-9 (PHQ-9), the 14-item Hamilton Anxiety Scale (HAMA), and 7-item Generalized Anxiety Disorder-7 (GAD-7).

The HAMD scale assesses a range of symptoms related to depression, such as guilt, suicidal thoughts, sleep disturbances, loss of interest, cognitive slowing, agitation, anxiety, physical symptoms, sexual issues, hypochondria, weight loss, insight, changes in symptoms throughout the day, depersonalization, paranoia, obsessive-compulsive behavior, feelings of impairment, despair, and inferiority. A score below 8 suggests normal functioning [13].

PHQ-9 reflects how the respondents were bothered by each symptom over the past two weeks. There are nine items, each of which corresponds to one of the nine Diagnostic and Statistical Manual of Mental Disorders (DSM) criteria for a major depressive episode [14]. PHQ-9 cut point > 4 was used to define depression [15, 16].

The HAMA scale consists of various symptoms such as tension, fear, sleep problems, cognitive issues, mood changes, muscle and sensory symptoms, as well as issues related to the cardiovascular, respiratory, gastrointestinal, genitourinary, and autonomic nervous systems, and behavioral performance during discussions. A score of less than 7 implies the absence of anxiety. [17].

The GAD-7 was used to evaluate the severity of anxiety symptoms. GAD-7 scores > 4 were used to define anxiety [15].

**PD rating scale**

The Unified Parkinson's Disease Rating Scale sponsored by the Movement Disorder Society (MDS-UPDRS) is the most commonly used scale for assessing PD and is supported by the MDS as the preferred tool for evaluating disability in PD [18, 19]. The MDS-UPDRS consists of four sections: I: Non-motor Experiences of Daily Living, II: Motor Experiences of Daily Living, III: Motor Examination, and IV: Motor Complications. Each participant had their total MDS-UPDRS score and scores for the four sections calculated [19].

**References:**

[1] Schulz KF, Altman DG, Moher D. CONSORT 2010 statement: updated guidelines for reporting parallel group randomized trials. Ann Intern Med. 2010;152:726-32.

[2] Haehner A, Tosch C, Wolz M, Klingelhoefer L, Fauser M, Storch A, et al. Olfactory training in patients with Parkinson's disease. PLoS One. 2013;8:e61680.

[3] Pekala K, Chandra RK, Turner JH. Efficacy of olfactory training in patients with olfactory loss: a systematic review and meta-analysis. Int Forum Allergy Rhinol. 2016;6:299-307.

[4] Doty RL, Tourbier I, Neff JK, Silas J, Turetsky B, Moberg P, et al. Influences of temporal lobe epilepsy and temporal lobe resection on olfaction. J Neurol. 2018;265:1654-65.

[5] Gudziol V, Lötsch J, Hähner A, Zahnert T, Hummel T. Clinical significance of results from olfactory testing. Laryngoscope. 2006;116:1858-63.

[6] Oleszkiewicz A, Bottesi L, Pieniak M, Fujita S, Krasteva N, Nelles G, et al. Olfactory training with Aromastics: olfactory and cognitive effects. Eur Arch Otorhinolaryngol. 2022;279:225-32.

[7] Wang P, Cai H, Luo R, Zhang Z, Zhang D, Zhang Y. Measurement of Cortical Atrophy and Its Correlation to Memory Impairment in Patients With Asymptomatic Carotid Artery Stenosis Based on VBM-DARTEL. Front Aging Neurosci. 2021;13:620763.

[8] Zhang Z-X, Zahner GEP, Román GC, Liu X-H, Wu C-B, Hong Z, et al. Socio-demographic variation of dementia subtypes in china: Methodology and results of a prevalence study in Beijing, Chengdu, Shanghai, and Xian. Neuroepidemiology. 2006;27:177-87.

[9] Katzman R, Zhang MY, Ouang Ya Q, Wang ZY, Liu WT, Yu E, et al. A Chinese version of the Mini-Mental State Examination; impact of illiteracy in a Shanghai dementia survey. J Clin Epidemiol. 1988;41:971-8.

[10] Yu J, Li J, Huang X. The Beijing version of the Montreal Cognitive Assessment as a brief screening tool for mild cognitive impairment: a community-based study. BMC Psychiatry. 2012;12:156.

[11] Damian AM, Jacobson SA, Hentz JG, Belden CM, Shill HA, Sabbagh MN, et al. The Montreal Cognitive Assessment and the mini-mental state examination as screening instruments for cognitive impairment: item analyses and threshold scores. Dement Geriatr Cogn Disord. 2011;31:126-31.

[12] Athey RJ, Porter RW, Walker RW. Cognitive assessment of a representative community population with Parkinson's disease (PD) using the Cambridge Cognitive Assessment-Revised (CAMCOG-R). Age Ageing. 2005;34:268-73.

[13] Zhao C, Cai H, Wang H, Ge Z. Correlation between serum renin-angiotensin system (RAS) level and depression and anxiety symptoms in patients with Parkinson's disease. Saudi J Biol Sci. 2021;28:2146-54.

[14] He C, Levis B, Riehm KE, Saadat N, Levis AW, Azar M, et al. The Accuracy of the Patient Health Questionnaire-9 Algorithm for Screening to Detect Major Depression: An Individual Participant Data Meta-Analysis. Psychother Psychosom. 2020;89:25-37.

[15] Lai J, Ma S, Wang Y, Cai Z, Hu J, Wei N, et al. Factors Associated With Mental Health Outcomes Among Health Care Workers Exposed to Coronavirus Disease 2019. JAMA Netw Open. 2020;3:e203976.

[16] Levis B, Benedetti A, Thombs BD. Accuracy of Patient Health Questionnaire-9 (PHQ-9) for screening to detect major depression: individual participant data meta-analysis. BMJ. 2019;365:l1476.

[17] Hamilton M. The assessment of anxiety states by rating. Br J Med Psychol. 1959;32:50-5.

[18] Goetz CG, Fahn S, Martinez-Martin P, Poewe W, Sampaio C, Stebbins GT, et al. Movement Disorder Society-sponsored revision of the Unified Parkinson's Disease Rating Scale (MDS-UPDRS): Process, format, and clinimetric testing plan. Mov Disord. 2007;22:41-7.

[19] Goetz CG, Tilley BC, Shaftman SR, Stebbins GT, Fahn S, Martinez-Martin P, et al. Movement Disorder Society-sponsored revision of the Unified Parkinson's Disease Rating Scale (MDS-UPDRS): scale presentation and clinimetric testing results. Mov Disord. 2008;23:2129-70.
